# Acute Kidney Injury in association with Acute Pancreatitis

**DOI:** 10.12669/pjms.343.14953

**Published:** 2018

**Authors:** Rubina Naqvi

**Affiliations:** 1 Prof. Dr. Rubina Naqvi, Professor of Nephrology, Sindh Institute of Urology and Transplantation (SIUT), Karachi, Pakistan

**Keywords:** Acute Kidney Injury (AKI), Acute pancreatitis, Mortality, Multi-organ Failure (MOF)

## Abstract

**Objective:**

To report case series of patients with acute kidney injury (AKI) developing in association with acute pancreatitis (AP).

**Methods:**

All patients coming to Sindh Institute of Urology and Transplantation (SIUT), who developed AKI in association with acute pancreatitis, were included in the study. AKI was defined with sudden rise in creatinine and decline in urine output with normal size kidneys and no previous co morbid. AP defined as patients with abdominal pain and > 3 times rise in serum amylase and /or lipase and radiology suggestive of AP.

**Results:**

Between 2000- 2017, 24 patients were found to have AKI in association with AP. Among these 13 were female, mean age was 36.875±10.152 years. Oligoanuria was present in 83%, 46% were icteric on presentation, 42% had circulatory failure, 33% had respiratory failure, 17% had abnormal Glasgow Coma Scale. Renal replacement was required in 79% patients. Complete renal recovery was observed in 54% patients, while 37% died during acute phase of illness. Multi-organ failure showed high prediction of mortality.

**Conclusion:**

Being a tertiary renal care unit only patients with renal failure were referred to our hospital, thus exact prevalence of AKI in AP is not known. Multi organ failure (MOF) has shown high mortality in this case series.

## INTRODUCTION

Acute pancreatitis; is acute inflammatory condition of pancreas, its presentation is variable from mild to severe with involvement to adjacent tissue to distant organs. Definitions and clinical presentations in different situations has been classified by International Working Committee and published in 1993.^1^ Multi organ failure (MOF) with severe form of acute pancreatitis can involve circulatory system with patients presenting with hypotension and shock, acute respiratory distress, hepatic involvement, gastro-intestinal involvement with bleeding, central nervous system involvement, coagulation abnormalities and renal failure.

The objective of present study was to look into details of medical records of patients presenting to a tertiary renal care unit with acute kidney injury/ acute renal failure in association with acute pancreatitis and discuss possible pathophysiological mechanisms and compare results with published reports on the subject.

## METHODS

This is a retrospective study done at Sindh Institute of Urology and Transplantation (SIUT), where case records of patients who presented with acute kidney injury in association with acute pancreatitis, were reviewed. Between year 2000 – 2017, twenty four such cases were found, their records were analyzed for associated multi organ involvement, duration of symptoms, severity of renal failure, course of illness and final outcome.

AKI was labeled when people with previous normal renal function developed deterioration of renal functions with rise in creatinine >2 mg/dl with decline in urinary output. If RIFLE criteria^2^ applied in retrospect all of these patients fall in category of ‘F’ or ‘L’ at time of reaching to our hospital.

Acute pancreatitis was diagnosed when patients presenting with abdominal pain, rise in serum amylase and / or lipase > 3 times of normal range and radiological findings suggestive of acute pancreatitis.

Circulatory failure was labeled when patients required inotropic support to achieve systolic blood pressure >100 mm of Hg. Respiratory failure when patient could not sustain oxygenation and required mechanical ventilation.

### Statistical Analysis

Values are expressed as mean ± standard deviation (SD) or percentage. Univariate analysis was performed on data employing the chi-square test. The student’s t test was used for continuous variables. Significance level was set at p <0.05.

## RESULTS

Of 24 studied patients 13 were females and rest male, with median age of 36 years in all patients. Most common symptom was abdominal/ epigastric pain which was present in all patients, followed by vomiting. Oligoanuria was reported in 20/24 (83.33%), 11/24 (45.83%) were icteric on presentation, 10/24 (41.66%) had circulatory failure, 8/24 (33.33%) had respiratory failure requiring mechanical ventilatory support, 4/24 (16.66%) had altered sensorium with disturbed Glasgow Coma Scale. Laboratory and other parameters of all patients on time of hospital admission are given in Table-I. Thrombocytopenia (platelet ≤150) in nine patients and thrombocytosis (platelet ≥ 350) seen in two patients. Eight patients had hyponatremia, four had hypokalemia and six hyperkalemia on presentation (Na < 129, K< 3.5 and >5.5 respectively). Hydration and antimicrobial therapy was given in all patients while renal replacement therapy provided in 19/24 (79.16%), another three patients died before renal replacement could started, all three of them had deranged coagulation and circulatory failure along with respiratory failure.

**Table-I:** Demography and laboratory data.

Parameter	Mean ± SD	Median	Range
Age in years	36.875±10.152	36	24-55
Days of insult	9.08±7.506	7	2-28
Hb g/dl	11.125±2.807	11	6.1-16.1
WBC x 10^9^/L	19.779±10.865	18.7	4.9-29.6
Platelet x 10^9^/L	214.083±135.528	187	36-659
Urea mg/L	242.666±124.573	212.5	79-571
Creatinine mg/L	11.102±7.572	9.25	2.88-39.24
Na meq/L	133.375±7.800	134.5	121-147
K meq/L	4.704±1.585	4.2	1.9-8.6
HCO3 meql/L	14.416±5.717	13.5	4-27
LDH 91-180 U/L	1978±1390.992	1540	213-4310
Amylase (25-125 U/L)	1837.708±1509.611	1205	273-5001
Lipase (0-60 U/L)	1358.6±1126.669	1013.5	204-4035
AST 10-40 U/L	242.416±400.979	94	19-1828
ALT 10-40 U/L	219.25±459.429	43.5	19-2210
Ca 8.4-10.2 mg/dl	5.86±0.924	5.7	5.4-8.2

Complete recovery from renal failure and resolution of pancreatic function was seen in 13/24 (54.16%) patients, 2/24 showed partial recovery till discharged from hospital and then lost to further follow up. A total of 9/24 (37.5%) died during acute phase of illness. Patients with coagulation failure, circulatory failure and respiratory failure revealed significantly high mortality. Comparison of characteristics among recovered and deceased patients is summarized in Table-II.

**Table-II:** Comparative Characteristics among Two groups.

Characteristics	Recovered (n=13)	Died (n=9)	P-value
Gender F/M	8/5	4/5	0.429
Age	35.84±9.29	40.60±9.58	0.270
Oligoanuria (%)	77	89	0.652
Days of insult	8.76±7.97	8±4.15	0.479
Hb g/dl	10.93±3.10	11.12±2.65	0.462
WBCx 10^9^/L	20.10±11.61	20.48±11.06	0.462
Platelets x10^9^/L	227.15±157.36	216.11±108.62	0.400
Urea mg/dl	254.07±138.69	236±119.67	0.341
Creatinine mg/dl	11.83±9.21	9.95±5.68	0.532
K meq/L	5.11±1.47	4.51±1.60	0.755
HCO3 meq/L	13±5.13	15.44±5.36	0.255
LDH U/L	1192.40±1271.24	1679.66±1223.20	0.395
Amylase U/L	1991.84±1378.48	1898.66±1802.65	0.400
Lipase U/L	1692.16±1387.70	868.33±250.58	0.342
ALT U/L	347.46±522.65	134±107.88	0.462
AST U/L	309.69±604.57	130±166.21	0.341
Number of Hemodialysis sessions	4.23±3.94	2.5±2.32	0.468
Deranged INR	3	7	0.032
Circulatory failure	2	8	<0.001
Required Mechanical Ventilator	1	9	<0.001

## DISCUSSION

Acute renal failure in association with acute pancreatitis has been reported as early as 1951, where 22 cases of “renal anoxia syndrome” have been reported; two of these were due to acute pancreatitis.^3^ Further search of literature found case reports and case series, which usually highlight high mortality in patients who develop acute renal failure in association with acute pancreatitis.^4^-^6^ The pathophysiological mechanisms are varied and controversial and so far reported include; inappropriate activation of trypsinogen to trypsin and thus undesired activation of digestive enzymes which cause pancreatic injury and can progress beyond pancreas to a systemic inflammatory response syndrome (SIRS), which can end up in MOF and death in many cases.^7^ Also reported are liberation of vasoactive substances,^8^ release of proteolytic enzymes from the damaged pancreas,^9^ and the presence of an intravascular coagulation.^10^

More recent literature describes role of hypoxemia, release of pancreatic amylase from the injured pancreas with resulting impairment of renal microcirculation, decrease in renal perfusion pressure due to abdominal compartment syndrome, intra-abdominal hypertension and hypovolemia.^11^

The activation of the complement system with severe forms of pancreatitis has also been reported, release of factor C5a occurs, which in turn stimulates macrophage and neutrophil recruitment. This further promotes intra-peritoneal inflammation and cytokine activation via transcription factors such as nuclear factor kappa B (NFκB). Both pro-inflammatory (tumor necrosis factor, interleukins IL-1, IL-6, IL-8 and platelet activating factor) and anti-inflammatory (interleukins IL-2, IL-10 and IL- 11) cytokines has been reported to play a role.^12^

The role of oxygen free radicals in promoting apoptosis in pancreatic acinar cell has also been studied.^13^ Animal study to examine the vascular reactivity of isolated mesenteric and pulmonary rings, after induced acute pancreatitis revealed that acute pancreatitis promotes reduction in the potency for Acetyl Choline in mesenteric artery that leads to marked increase in nitrite/nitrate (NO) levels. Severe tissues damage and endothelial dysfunction is described to be strongly linked to overproduction of NO.^14^

From Pakistan; AKI as complication of AP has been variably reported 1-14%, while mortality with AP ranges from 0 to 30%.^15^-^18^ Present study is based on data from a tertiary renal care hospital therefore; prevalence of AP or AKI with AP cannot be commented as AP was treated by internists and surgeons according to etiology and only referred here when renal dysfunction is noticed. Tran DD et al. report 16% prevalence of AKI with AP^19^, same authors have reported better prognosis in nonoliguric form of renal failure in their AP patients, but in our study 83% patients were oliguric on presentation with closely falling frequency among patients who recovered and died during acute illness. All the patients in our study were young with median age of 36 years thus adverse outcome related to advancing age cannot be commented as old age is generally taken as adverse factor for poor outcome. Similarly gender distribution in our studied population is 1.8:1(F:M) and no significant effect on outcome has been observed. Delayed organ specific management can affect patient prognosis and in our part of world, especially in our country Pakistan lack of health care facilities and disparity in resources availability often guides the fortune. In present study patients reached to this facility ranging from two to 28 days of developing the symptoms but this duration was not significantly different among survived and deceased patients.

Leucopenia has been reported previously in patients with MOF with background AP^19^, but we have not experienced so, 83% of our patients had high total leukocyte count while rest had within normal range. Thrombocytopenia if seen had identical prevalence among two groups.

Renal replacement therapy was desired in 92% of our patients, three of our patients died even before getting stability to start hemodialysis. We do not have facility of continuous renal replacement which could be a better option in hemodynamically unstable patients of this series. Tran DD et al. have reported 100% mortality in patients requiring hemodialysis;^19^ our findings differ from them as we see 37.5% deaths among our studied population.

### Limitation of study

Being a tertiary renal care unit we only see patients who had been referred here because of renal failure and mainly for reason of non affordability of health care at private set-up. Many patients with renal failure might have been sick enough to get transferred here for renal care. Thus prevalence of AKI in association with AP or mortality in patients with MOF cannot be exactly judged with this case series. Also during previous years there was no facility of continuous renal replacement and thus patients with resistant circulatory failure could not get benefit of renal replacement therapy even after reaching to renal unit.

At tertiary renal care units only observational studies can be done with available results of patient’s records. To know exact prevalence of AKI associated with AP in country, multi center studies at national level should be planned, so that exact intensity of problem, associated factors, management availabilities and outcome can be addressed in a better way.

## CONCLUSIONS

Being a tertiary renal care unit only patients with renal failure were referred to our hospital, thus exact prevalence of AKI in AP is not known. Multi organ failure (MOF) has shown high mortality in this case series.
